# Association of *MTHFR* C677T and A1298C Polymorphisms with First-Episode Myocardial Ischemia: A Case–Control Study

**DOI:** 10.3390/genes16080858

**Published:** 2025-07-23

**Authors:** Iulia Andreea Badea, Lavinia Carmen Daba, Nicoleta Leopa, Irinel Raluca Parepa, Sorina Ispas, Mihaela Botnarciuc

**Affiliations:** 1 Faculty of Medicine, Ovidius University, 900470 Constanta, Romania; iulia.badea@365.univ-ovidius.ro (I.A.B.); lavinia.daba@univ-ovidius.ro (L.C.D.); irinel_parepa@yahoo.com (I.R.P.); sorina.ispas@365.univ-ovidius.ro (S.I.); 2 Department of General Surgery, Emergency Hospital of Constanța, 900591 Constanta, Romania; mihaela.botnarciuc@univ-ovidius.ro; 3 Department of Cardiology, Emergency Hospital of Constanța, 900591 Constanta, Romania; 4 Blood Transfusions Unit, Emergency Hospital Constanta, 900591 Constanta, Romania

**Keywords:** *MTHFR* polymorphism, C677T, A1298C, myocardial ischemia, genetic susceptibility

## Abstract

Background: Myocardial ischemia remains a major cause of morbidity and mortality worldwide. Although traditional risk factors are well-established, genetic predisposition—particularly involving *MTHFR* polymorphisms—has garnered increasing attention. This study investigates the association between *MTHFR* C677T and A1298C polymorphisms and first-episode myocardial ischemia in a Romanian population. Methods: This study included 69 adult patients with first-episode myocardial ischemia and 55 healthy controls, matched by age and sex. Participants were recruited from southeastern Romania between 2023 and 2025. Clinical data—such as blood pressure, body mass index, smoking, and alcohol consumption—were recorded. Genotyping for *MTHFR* C677T and A1298C polymorphisms was performed using a real-time PCR-based assay (Bosphore^®^ *MTHFR* 677-1298 Detection Kit v2), following the manufacturer’s protocol. Results: A significantly higher frequency of homozygous mutant genotypes was observed in patients with myocardial ischemia. The TT genotype of *MTHFR* C677T was present in 71% of patients, compared to only 7.3% of controls. Similarly, the CC genotype of A1298C was detected in 59.4% of patients, versus 7.3% in controls. These genotypic patterns suggest a strong genetic predisposition among affected individuals. The association between *MTHFR* polymorphisms and myocardial ischemia was particularly evident in participants over 50 years of age, indicating a possible interaction between genetic vulnerability and age-related cardiovascular risk. Conclusions: Our findings indicate a strong association between *MTHFR* C677T and A1298C homozygous mutant genotypes and the risk of first-episode myocardial ischemia, particularly in older adults. These results underscore the potential role of genetic screening in early cardiovascular risk stratification.

## 1. Introduction

Myocardial ischemia (MI) remains a leading cause of morbidity and mortality worldwide, representing a major public health burden despite advances in preventive and interventional cardiology [1,2]. It is characterized by an imbalance between myocardial oxygen supply and demand, typically due to obstructive coronary artery disease, and may present clinically as angina pectoris, ECG changes, or myocardial injury with or without infarction [3,4].

While traditional cardiovascular risk factors—such as hypertension, dyslipidemia, obesity, smoking, and diabetes—are well-established contributors to MI, growing evidence supports a role for genetic predisposition in modulating individual susceptibility [5]. Among the genes implicated, methylenetetrahydrofolate reductase (*MTHFR*) is of particular interest due to its involvement in folate metabolism and homocysteine regulation [6]. The *MTHFR* enzyme catalyzes the conversion of 5,10-methylenetetrahydrofolate to 5-methyltetrahydrofolate, a co-substrate for homocysteine remethylation to methionine [7]. Genetic variants of the *MTHFR* gene, notably C677T and A1298C, can impair enzyme function, leading to hyperhomocysteinemia, a known prothrombotic and atherogenic factor associated with increased cardiovascular risk [8,9].

The C677T variant results in a thermolabile enzyme with reduced activity, especially in homozygous TT individuals, while the A1298C variant is associated with decreased enzymatic activity but to a lesser extent [10]. The role of these polymorphisms in cardiovascular disease has been widely studied, though findings remain inconclusive across populations, with varying effects based on ethnicity, age, and environmental factors [11].

In this context, we aimed to investigate the association between *MTHFR* C677T and A1298C polymorphisms and the risk of first-episode myocardial ischemia in a Romanian population. By comparing genotypic distributions between MI patients and matched healthy controls, we sought to clarify the contribution of these genetic variants to early-onset ischemic events.

## 2. Material and Methods

### 2.1. Study Group

A case–control study was conducted between February 2023 and February 2025 at the Cardiology Clinic of the Constanța County Emergency Hospital, Romania. The study included 69 adult patients diagnosed with first-episode MI, prospectively admitted and managed during the study period. Control subjects (*n* = 55) were selected from the general population of the same geographic area and were matched to cases based on age and sex distribution. Controls were defined as individuals with no history of cardiovascular or chronic disease and a normal clinical exam, indicating an overall healthy status. Control participants were selected based on clinical examination, absence of known chronic disease or medication, and normal findings on routine preventive screening. Blood pressure measurements, including DBP, fell within accepted physiological norms and were not suggestive of subclinical hypertension or cardiovascular pathology.

According to the European Society of Cardiology guidelines, MI refers to a state of insufficient blood flow and oxygen delivery to the myocardium, usually due to obstructive coronary artery disease. It may be asymptomatic or present clinically as chest pain (angina), ECG changes, or regional wall motion abnormalities on cardiac imaging [1].

Inclusion criteria for the study group were: (1) adults aged ≥18 years, (2) first-time diagnosis of myocardial ischemia (confirmed by clinical, ECG, and imaging criteria), (3) admitted between February 2023 and February 2025, and (4) completed informed consent. Exclusion criteria for both groups were as follows: (1) history of myocardial infarction, (2) previous cardiac or vascular surgery, (3) chronic kidney disease, malignancy, or autoimmune conditions, (4) incomplete clinical/genetic data, and (5) refusal to participate.

### 2.2. Data Collection

Patients were consecutively enrolled upon admission, and control subjects were selected through voluntary enrollment during routine medical check-ups.

All patients with ischemic lesions received standard pharmacological therapy and medical–cardiological supervision according to institutional protocols. Demographic and clinical data included age, gender, and alcohol consumption and smoking status (according to the National Institute on Alcohol Abuse and Alcoholism and the Center for Disease Control and Prevention) of the participants were recorded, and the body mass index (BMI) was calculated [12,13]. Hardy–Weinberg Equilibrium (HWE) testing was performed exclusively in the control group using the chi-square goodness-of-fit test. This approach is standard in genetic epidemiology, as HWE assessment in case groups is not appropriate due to potential distortion of genotype frequencies by disease-related selection.

### 2.3. Genotyping

Genotyping of the *MTHFR* gene polymorphisms C677T (rs1801133) and A1298C (rs1801131) was performed using the Bosphore^®^ *MTHFR* 677-1298 Detection Kit v2 (Anatolia Geneworks, Sultanbeyli/İstanbul, Turkey), based on real-time PCR (qPCR) technology. The kit employs fluorescent allele-specific probes and internal controls, validated according to CE-IVD standards. This multiplex assay allows simultaneous detection of wild-type and mutant alleles for both loci in a single reaction.

Whole blood samples (10 µL) were incubated with EX-Tract DNA solution, a proprietary lysis reagent included in the kit, at 80 °C for 15–20 min. This step ensured direct DNA release without the need for separate DNA extraction. After lysis, the sample lysate was diluted and used directly for PCR amplification. The PCR mix (20 µL total volume) included 15 µL PCR Master Mix and 5 µL of prepared sample or control. Amplification was carried out on a compatible real-time PCR platform (e.g., Bio-Rad, Hercules, CA, USA. CFX96), following the manufacturer’s cycling conditions: initial denaturation at 95 °C for 14 min and 30 s, followed by 45 cycles of denaturation at 95 °C for 30 s, and annealing/extension at 64 °C for 60 s with data acquisition.

PCR was run using allele-specific fluorescent probes: FAM (C677T mutant), HEX (C677T wild-type), Texas Red (A1298C mutant), and Cy5 (A1298C wild-type). While genotyping was performed using a CE-IVD validated real-time PCR assay with built-in quality controls, the lack of independent validation via Sanger sequencing represents a technical limitation of the present study. Further sequencing-based confirmation is planned in future expansions of this work. A heterozygous positive control was included in each run to verify assay performance. Genotypes were interpreted based on signal presence in the respective fluorescence channels, following the manufacturer’s interpretation chart. Only results meeting internal quality control parameters were considered valid.

### 2.4. Study Endpoints

*Primary Endpoint* was to evaluate the association between *MTHFR* C677T and A1298C gene polymorphisms and the risk of first-episode MI by comparing the genotype distribution (TT, CT, and CC for C677T and AA, AC, and CC for A1298C) between patients with MI and matched healthy controls.

### 2.5. Secondary Endpoints

To assess the influence of *MTHFR* genotypes on cardiovascular risk factors (e.g., blood pressure, BMI, smoking status) in MI patients compared to controls.

To determine whether the association between *MTHFR* polymorphisms and MI risk is age-dependent, stratified analysis of participants below and above 50 years of age was conducted.

### 2.6. Ethical Consideration

The study protocol was approved by the Local Ethics Committee of Constanța County Emergency Hospital (approval no. 27/10 January 2023), and all participants provided written informed consent. The study was conducted in accordance with the Declaration of Helsinki.

### 2.7. Statistical Analysis

The characteristics of the study participants were described using descriptive statistics. Using the mean and standard deviation, quantitative variables were described. Meanwhile, frequencies and percentages were used to summarize qualitative variables. Using Pearson’s Chi-square test and HWE, the relationship between illness status and genetic variations was examined. Using odds ratios (ORs) and 95% confidence intervals (CIs), the corresponding ꭓ^2^ distribution test was computed. Age and BMI adjustments were made before conducting association analyses using multivariate logistic regression analysis. The least significant difference test and one-way analysis of variance were used to compare clinical parameters of various genotypes between MI patients and healthy controls. A *p*-value < 0.05 was considered statistically significant. The SPSS statistical software package for Windows version 28.0 (IBM, Armonk, NY, USA) was used for all statistical analyses.

## 3. Results

The comparative analysis between MI patients and controls revealed several significant differences in clinical profiles (Table 1). Although the mean age was similar between groups (61.8 ± 10.99 vs. 63.9 ± 10.89 years, *p* = 0.126) and sex distribution did not reach statistical significance (*p* = 0.098), notable differences emerged in cardiovascular parameters. MI patients had significantly higher systolic (139.41 ± 34.72 vs. 119.65 ± 2.11 mmHg, *p* = 0.044) and diastolic blood pressure (69.21 ± 14.25 vs. 54.42 ± 1.01 mmHg, *p* = 0.013). Regarding BMI, patients with MI showed a significantly higher frequency of being overweight and obese (43.5% and 15.9%, respectively), whereas the control group had a predominance of normal weight (76.4%) and no cases of obesity (*p* = 0.001). Differences in smoking and alcohol use were not statistically significant but trended higher in the MI group.

Allele frequencies for the C677T polymorphism in the control group were 83.6% for the C allele and 16.4% for the T allele. The genotype distribution was CC = 41, CT = 10, and TT = 4. The Hardy–Weinberg Equilibrium test revealed a slight deviation (χ^2^ = 6.20, *p* = 0.045) (Table 2).

The *MTHFR* C677T polymorphism showed a strong association with MI (Table 3). Among MI patients, the most frequent genotype was TT (71%), followed by CT (18.8%) and CC (10.1%). In contrast, the control group showed a predominance of the CC genotype (74.5%), with very low frequencies of TT (7.3%) and CT (18.2%). In the allelic/genotypic model, the CT genotype conferred a significantly increased MI risk with an OR of 8.10 [1.72–38.19], *p* = 0.008, and the CC genotype presented a dramatically increased risk with an OR of 44.19 [9.68–201.64], *p* < 0.001, compared to the TT genotype. In the dominant model (TT vs. CT + CC), combined CT + CC genotypes were associated with a reduced odds of MI (OR = 0.142 [0.029–0.691], *p* = 0.016), indicating that the TT genotype may be the risk variant. In the recessive model (TT + CT vs. CC), the CC genotype showed a strong association with increased risk (OR = 0.158 [0.036–0.700], *p* = 0.015), further reinforcing the contribution of this locus to MI susceptibility.

For the A1298C polymorphism, the most frequent genotype among MI patients was CC (59.4%), with AC (24.6%) and AA (15.9%) being less common. In the control group, AA (83.6%) was predominant, while CC (7.3%) and AC (9.1%) were rare. The AA genotype was significantly associated with increased MI risk (OR = 27.28 [6.13–121.33], *p* < 0.001). In the recessive model, AA carriers had a significantly higher risk compared to CC + AC (OR = 0.096 [0.020–0.458], *p* = 0.003). The dominant model (AA + AC vs. CC) did not show a significant association (*p* = 0.292), indicating that homozygosity may be critical in the pathogenicity of this SNP. The A1298C polymorphism showed a similar trend. While the AC genotype was not significantly associated with MI (*p* = 0.176), the AA genotype had a strong association with disease (*OR* = 27.28 [6.13–121.33], *p* < 0.001). The recessive model (AA vs. CC + AC) showed significant risk elevation (*OR* = 0.096 [0.020–0.458], *p* = 0.003), confirming the role of homozygosity in disease susceptibility.

The role of genetic variants was further explored in relation to age (Table 4). Among patients aged ≥50 years, a clear association was observed between both polymorphisms and MI. For the C677T genotype, individuals ≥ 50 years carrying the CT or CC genotypes had a significantly lower frequency in the control group compared to MI patients, with an OR = 0.041 [0.011–0.151], *p* < 0.001. However, in those under 50 years, this association was not statistically significant, suggesting that the effect of *MTHFR* variants may be age-dependent, possibly due to cumulative vascular damage or interaction with other risk factors over time.

Similarly, for the A1298C genotype, among subjects ≥ 50 years, those with AC or AA genotypes were at significantly increased risk of MI compared to controls (OR = 0.070 [0.018–0.269], *p* < 0.001). No significant associations were found in individuals < 50 years, again highlighting a potential synergistic effect of age and genetic predisposition.

## 4. Discussion

Our study demonstrates a significant association between *MTHFR* gene polymorphisms (C677T and A1298C) and the risk of first-episode myocardial ischemia in a southeastern Romanian population. We found that the TT genotype of C677T and CC genotype of A1298C were markedly more frequent in MI patients compared to controls, with high odds ratios, especially in individuals aged ≥50 years. These results are consistent with and expand upon previous studies that highlight the role of *MTHFR* polymorphisms in cardiovascular risk, though with notable population-specific differences [14].

In our cohort, the TT genotype for C677T was found in 71% of MI patients and only 7.3% of controls, with an OR of 44.19, while the CC genotype for A1298C appeared in 59.4% of patients and 7.3% of controls (OR = 27.28). These findings strongly support a genetic predisposition to MI conferred by homozygous mutant genotypes, especially in older individuals. These results are in line with the study by Zhao et al. [15] in a Chinese population, which reported that the TT genotype of C677T was significantly associated with coronary artery disease, particularly in individuals with high homocysteine levels. Similarly, Refsum et al. [16] found a consistent link between hyperhomocysteinemia and cardiovascular risk in individuals with *MTHFR* mutations, highlighting the biological plausibility of our findings. In an Iranian population, Chen et al. [17] found that TT genotype carriers had a 2.6-fold increased risk of ischemic heart disease compared to those with CC genotype, though with lower frequency values than in our population. A meta-analysis of 40 studies concluded that the effect of *MTHFR* C677T on coronary heart disease risk was more pronounced in low-folate populations, which likely includes southeastern Europe [18,19].

With respect to A1298C, our results confirm the literature findings that the homozygous CC genotype was associated with increased risk of MI, especially when combined with smoking and high LDL levels [20] Our data align more closely with studies conducted in Eastern Europe and South Asia, which found a significant association between combined *MTHFR* C677T/A1298C genotypes and early-onset myocardial infarction, emphasizing a synergistic effect when both mutations are present [21]. Interestingly, in the present study, heterozygous genotypes (CT and AC) showed only intermediate or statistically insignificant risk, supporting the recessive inheritance pattern suggested by Sibani et al. [22], who found that only homozygous mutant carriers experienced significant reduction in *MTHFR* enzyme activity.

The age-stratified analysis adds a novel dimension to our findings. We observed that both polymorphisms had a stronger association with MI in individuals aged ≥50 years, while no significant relationship was detected in the younger subgroup. This supports the hypothesis proposed that the pathogenic consequences of *MTHFR* mutations intensify with age, possibly due to cumulative oxidative and endothelial damage [23]. Few studies have reported such high ORs as ours; however, this may be attributed to our stringent inclusion of first-episode MI patients, reducing confounding from recurrent ischemia or post-surgical pathology, and the high prevalence of the TT and CC genotypes in the Romanian cohort [24]. Moreover, the control group was carefully selected to exclude individuals with any known cardiovascular disease or related metabolic conditions, which may have increased the contrast between groups.

Moreover, emerging evidence highlights the importance of gene–environment interactions in modulating the effect of *MTHFR* polymorphisms [19]. In particular, folate status and B-vitamin (B6, B12) intake play crucial roles in homocysteine metabolism, which is the primary biological pathway linking *MTHFR* mutations to endothelial dysfunction and atherothrombosis [11,17]. For instance, individuals with the TT genotype for C677T may only exhibit elevated homocysteine levels and increased cardiovascular risk in the context of low folate or vitamin B12 intake [19]. Conversely, adequate dietary intake or supplementation may attenuate the genotype-associated risk.

## 5. Strengths and Limitations

This study has several notable strengths. First, it focused exclusively on first-episode myocardial ischemia, eliminating confounding factors from recurrent ischemic events or prior interventions and ensuring a more accurate assessment of genetic risk at initial presentation. Second, the genotyping was performed using a validated, high-sensitivity real-time PCR assay, with internal positive controls, ensuring reliable and reproducible detection of both *MTHFR* C677T and A1298C polymorphisms. Third, the inclusion of a well-defined control group, matched for age and sex and free from known cardiovascular disease, allowed for meaningful comparison. Additionally, the use of age-stratified analysis provided novel insight into the interaction between genetic predisposition and aging. Finally, this is one of the few studies conducted in a southeastern European population, contributing valuable data to a region with limited genetic epidemiology research related to myocardial ischemia.

Despite the strengths of our study, some limitations must be acknowledged. A major limitation of the present study is the lack of plasma homocysteine level measurements, which would have provided functional correlation between *MTHFR* polymorphisms and metabolic consequences such as hyperhomocysteinemia. However, the assessment of homocysteine concentrations is part of a separate ongoing study and will be reported independently. As such, this current analysis is focused exclusively on the genotypic distribution and its association with myocardial ischemia risk, without linking it directly to biochemical pathways.

## 6. Conclusions

In our study, we identified a strong association between *MTHFR* C677T and A1298C polymorphisms and the risk of first-episode myocardial ischemia in a southeastern Romanian population. The high frequency of homozygous mutant genotypes among myocardial ischemia patients highlights a possible genetic predisposition, particularly in individuals over 50. These findings support the relevance of targeted genetic investigation in early cardiovascular risk evaluation.

## Figures and Tables

**Table 1:** Characteristics of patients with myocardial ischemia and the controls.

Variable	Patients with MI (*n* = 69)	Controls (*n* = 55)	*p*-Value
Age (years), mean ± SD	61.83 ± 10.99	63.87 ± 10.89	0.126
˂50 years	11 (15.9)	9 (16.4)	
50–59 years	13 (18.8)	12 (21.8)	
60–69 years	30 (43.5)	15 (27.3)	
70–79 years	13 (18.8)	14 (25.5)	
>80 years	2 (2.9)	5 (9.1)	
Sex			0.098
Male	30 (43.5)	16 (29.1)	
Female	39 (56.5)	39 (70.9)	
Smoking	22 (31.9)	12 (21.8)	0.209
Habitual alcohol use	5 (7.2)	10 (18.2)	*0.063*
SBP, mmHg	139.41 ± 34.72	119.65 ± 2.11	*0.044*
DBP, mmHg	69.21 ± 14.25	54.42 ± 1.01	*0.013*
BMI, kg/m^2^	25.6 ± 3.97	23.72 ± 1.07	*0.001*
Underweight	10 (14.4)	0	
Normal weight	18 (26.1)	42 (76.4)	
Overweight	30 (43.5)	13 (23.6)	
Obesity	11 (15.9)	0	

SD, standard deviation; SBP, systolic blood pressure; DBP, diastolic blood pressure; and BMI, body mass index. Number of cases, with percentages in parenthesis. Values in italics indicate statistical significance (*p* < 0.050).

**Table 2:** C677T and A1298C genotype and allele frequencies with Hardy–Weinberg analysis for the controls.

Polymorphism	Group	Allele	Count	Frequency (%)	HWE *p*-Value (Controls)
C677T	MI patients	C	27	19.6	-
		T	111	80.4	-
	Controls	C	92	83.6	*0.045*
		T	18	16.4	
A1298C	MI patients	A	39	28.3	-
		C	99	71.7	-
	Controls	A	97	88.2	*0.001*
		C	13	11.8	

HWE, Hardy–Weinberg Equilibrium and MI, myocardial ischemia. Values in italics indicate statistical significance (*p* < 0.050).

**Table 3:** Multivariate analysis of association between the polymorphism of *MTHFR* C677T, A1298C, and risk of myocardial ischemia.

Variable		Patients with MI (*n* = 69)	Controls (*n* = 55)	OR [95% CI]	*p*-Value
**C677T**	Genotype				
	TT	49 (71)	4 (7.3)	-	-
	CT	13 (18.8)	10 (18.2)	8.098 [1.717–38.189]	*0.008*
	CC	7 (10.1)	41 (74.5)	44.186 [9.683–201.639]	*˂0.001*
	Dominant				
	TT	49 (71)	4 (7.3)	(Reference)	-
	CC + CT	20 (29)	51 (92.7)	0.142 [0.029–0.691]	*0.016*
	Recessive				
	TT + CT	62 (89.9)	14 (25.5)	(Reference)	-
	CC	7 (10.1)	41 (74.5)	0.158 [0.036–0.700]	*0.015*
A1298C	Genotype				
	CC	41 (59.4)	4 (7.3)	-	-
	AC	17 (24.6)	5 (9.1)	3.353 [0.582–19.312]	0.176
	AA	11 (15.9)	46 (83.6)	27.275 [6.131–121.334]	*˂0.001*
	Dominant				
	CC	41 (59.4)	4 (7.3)	(Reference)	-
	AA + AC	28 (40.6)	51 (92.7)	0.377 [0.062–2.310]	0.292
	Recessive				
	CC + AC	58 (84.1)	9 (16.4)	(Reference)	-
	AA	11 (15.9)	46 (83.6)	0.096 [0.020–0.458]	*0.003*

Variables are expressed as number of cases, with percentages in parenthesis. Values in italics indicate statistical significance (*p* < 0.050). CRC, colorectal cancer; OR, odds ratio; and CI, confidence interval.

**Table 4:** Multivariate analysis of the association between *MTHFR* C677T, A1298C, and risk of myocardial ischemia stratified by age.

Genotype	Age (Years)	Patients with MI (*n* = 69)	Controls (*n* = 55)	OR [95% CI]	*p*-Value
C677T					
TT	<50	6 (8.7)	0	-	-
CT + CC	<50	5 (7.3)	9 (16.4)	-	NS
TT	≥50	43 (62.3)	4 (7.3)	(Reference)	-
CT + CC	≥50	15 (21.7)	42 (76.3)	0.041 [0.011–0.151]	*˂0.001*
A1298C					
CC	<50	4 (5.8)	0	-	-
AC + AA	<50	7 (10.2)	9 (16.4)	-	NS
CC	≥50	37 (53.6)	4 (7.3)	(Reference)	-
AC + AA	≥50	21 (30.4)	42 (76.3)	0.070 [0.018–0.269]	*˂0.001*

Variables are expressed as number of cases, with percentages in parenthesis. Values in italics indicate statistical significance (*p* < 0.050). OR, odds ratio; CI, confidence interval; and NS, non-significant.

## Data Availability

The original contributions presented in this study are included in the article. Further inquiries can be directed to the corresponding authors.

## Abbreviations

The following abbreviations are used in this manuscript:

MI	myocardial ischemia
*MTHFR*	methylenetetrahydrofolate reductase
BMI	body mass index
